# Building and Breaking Bonds by Homogenous Nucleation in Glass-Forming Melts Leading to Transitions in Three Liquid States

**DOI:** 10.3390/ma14092287

**Published:** 2021-04-28

**Authors:** Robert F. Tournier, Michael I. Ojovan

**Affiliations:** 1 LNCMI-EMFL, CNRS, Université Grenoble Alpes, INSA-T, UPS, 38042 Grenoble, France; 2 Department of Materials, Imperial College London, London SW7 2AZ, UK; m.ojovan@imperial.ac.uk; 3 Department of Radiochemistry, Lomonosov Moscow State University, 119991 Moscow, Russia

**Keywords:** liquid–liquid transitions, glass phase, amorphous, undercooling, superheating, percolation threshold, microheterogeneity

## Abstract

The thermal history of melts leads to three liquid states above the melting temperatures T_m_ containing clusters—bound colloids with two opposite values of enthalpy +Δε_lg_ × ΔH_m_ and −Δε_lg_ × ΔH_m_ and zero. All colloid bonds disconnect at T_n+_ > T_m_ and give rise in congruent materials, through a first-order transition at T_LL_ = T_n+_, forming a homogeneous liquid, containing tiny superatoms, built by short-range order. In non-congruent materials, (T_n+_) and (T_LL_) are separated, T_n+_ being the temperature of a second order and T_LL_ the temperature of a first-order phase transition. (T_n+_) and (T_LL_) are predicted from the knowledge of solidus and liquidus temperatures using non-classical homogenous nucleation. The first-order transition at T_LL_ gives rise by cooling to a new liquid state containing colloids. Each colloid is a superatom, melted by homogeneous disintegration of nuclei instead of surface melting, and with a Gibbs free energy equal to that of a liquid droplet containing the same magic atom number. Internal and external bond number of colloids increases at T_n+_ or from T_n+_ to T_g_. These liquid enthalpies reveal the natural presence of colloid–colloid bonding and antibonding in glass-forming melts. The Mpemba effect and its inverse exist in all melts and is due to the presence of these three liquid states.

## 1. Introduction

Glass-forming melt transformations have been mainly studied, for many years, around the glass transition temperature T_g_ and sometimes up to the liquidus temperature T_liq_. The liquid properties are often neglected because the classical nucleation equation predicts the absence of growth nuclei and nucleation phenomenon above the melting temperature. The presence of growth nuclei above T_m_ being known [1,2,3], an additional enthalpy is added to this equation to explain these observations. A new model of nucleation is built from the works of Turnbull’s [4] characterized by two types of homogeneous nucleation temperatures below and above T_m_. The new additional enthalpy is a quadratic function of the reduced temperature θ = (T − T_m_)/T_m_ as shown by a revised study of the maximum undercooling rate of 38 liquid elements using Vinet’s works [5,6]. A concept of two liquids is later introduced to explain the glass phase formation at T_g_ by an enthalpy decrease from liquid 1 to liquid 2 at this temperature. New laws minimizing the numerical coefficients of each quadratic equation are established determining the enthalpies ε_ls_(0) × ΔH_m_ of liquid 1 and ε_gs_(0) × ΔH_m_ of liquid 2 for each θ value, with ΔH_m_ being the melting enthalpy [7,8]. The thermodynamic transition at T_g_ is characterized by a second-order phase transition and a heat capacity jump defined by the derivative of the difference (ε_ls_(θ) − ε_gs_(θ)) ΔH_m_ which is equal to 1.5 ΔS_m_ for many glass transitions with ΔS_m_ being the melting entropy [9].

The glass transition results from the percolation of superclusters formed during cooling below T_m_ [10,11,12]. A thermodynamic transition characterized by critical parameters occurs by breaking bonds (configurons) and when the percolation threshold of configurons is attained [13,14,15,16,17]. Building bonds by enthalpy relaxation below T_g_ has for consequence the formation of a hidden undercooled phase called phase 3 with an enthalpy (ε_ls_(θ) − ε_gs_(θ)) ΔH_m_ equal to that of configurons with a residual bond fraction which can be overheated up to T_n+_ > T_m_ before being melted [18]. The homogeneous nucleation temperature at T_n+_ occurs in overheated liquids and is predicted for many molecular and metallic glass-forming melts.

This paper is devoted to phase transitions above T_m_ completing our recent work, showing that the dewetting temperatures of prefrozen and grafted layers in ultrathin films are equal to T_n+_ [19]. The latent heats are exothermic or endothermic without knowing the explanation. The existence of a first-order transition is claimed for Pd_42.5_Ni_42.5_P_15_ and La_50_Al_35_Ni_15_ liquid alloys [20,21]. Our nucleation model of melting the liquid mean-range order by breaking residual bonds predicted all values of T_n+_ and exothermic enthalpies at this temperature. The observation of endothermic latent heats showed the existence of three liquid states at T_m_, the first one with a positive enthalpy ε_gs_(0) × ΔH_m_, the second one zero, and the third one −ε_gs_(0) × ΔH_m_, which is negative. The liquid is homogeneous above T_n+_ when its enthalpy is equal to zero. The existence of various liquid states was also predicted without using a non-classical nucleation equation [22]. The formation temperature of a homogeneous liquid state was observed by measuring the density or the viscosity during heating and cooling, determining the point where the branching of these quantities disappears. Colloidal states were observed below this homogenization temperature and composed of thousands of atoms defining liquid heterogeneities [23,24,25,26,27]. Our objectives were to predict all these phase transitions.

## 2. The Homogeneous Nucleation

The Gibbs free energy change for a nucleus formation in a melt was given by Equation (1) [6,9]:

(1) $$\Delta G_{\mathrm{ls}}=\left(\theta -\varepsilon_{\mathrm{ls}}\right)\Delta H_{m}/V_{m}\times 4{\pi R}^{3}/3+4{\pi R}^{2}\sigma_{\mathrm{ls}}$$

where R is the nucleus radius and following Turnbull [4], σ_ls_ its surface energy, given by Equation (2), θ the reduced temperature (T − T_m_)/T_m_, ΔH_m_ the melting enthalpy at T_m_, and V_m_ the molar volume:

(2) $$\sigma_{\mathrm{ls}}{\left(V_{m}/N_{A}\right)}^{-1/3}={\;\alpha }_{\mathrm{ls}}\Delta H_{m}/V_{m}$$

A complementary enthalpy −ε_ls_ × ΔH_m_/V_m_ was introduced, authorizing the presence of growth nuclei above T_m_. The classical nucleation equation was obtained for ε_ls_ = 0.

The critical radius $R_{\mathrm{ls}}^{*}$ in Equation (3) and the critical thermally activated energy barrier $\frac{\Delta G_{\mathrm{ls}}^{*}}{k_{B}T}$ in Equation (4) are calculated assuming dε_ls_/dR = 0:

(3) $$R_{\mathrm{ls}}^{*}=\frac{-2\alpha_{\mathrm{ls}}}{\left(\theta -\varepsilon_{\mathrm{ls}}\right)}{\left(\frac{V_{m}}{N_{A}}\right)}^{-1/3}$$

(4) $$\frac{\Delta G_{\mathrm{ls}}^{*}}{k_{B}T}=\frac{16\pi \Delta S_{m\;}\alpha_{\mathrm{ls}}^{3}}{3N_{A}k_{B}\left(1+\theta \right){\left(\theta -\varepsilon_{\mathrm{ls}}\right)}^{2}}$$

These critical parameters are not infinite at the melting temperature T_m_ because ε_ls_ is not equal to zero. The nucleation rate J = K_v_exp(−$\frac{\Delta G_{\mathrm{ls}}^{*}}{k_{B}T}$) is equal to 1 when Equation (5) is respected:

(5) $$\Delta G_{\mathrm{ls}}^{*}/k_{B}T=\ln\left(K_{v}\right)$$

The surface energy coefficient α_ls_ in Equation (2) is determined from Equations (4) and (5) and given by Equation (6):

(6) $$\alpha_{\mathrm{ls}}^{3}=\frac{3N_{A}k_{B\;}\left(1+\theta \right){\left(\theta -\varepsilon_{\mathrm{ls}}\right)}^{2}}{16\pi \Delta S_{m}}\ln\left(K_{v}\right)$$

The nucleation temperatures θ_n_ obtained for d$\alpha_{\mathrm{ls}}^{3}$/dθ = 0 obeys (7):

(7) $${d\alpha }_{\mathrm{ls}}^{3}/d\theta \;\sim (\theta_{n+}-\varepsilon_{\mathrm{ls}})\left(3\theta_{n-}+2-\varepsilon_{\mathrm{ls}}\right)=0$$

In addition to the nucleation temperature T_n-_ below T_m_, the existence of homogeneous nucleation up to T_n+_ above T_m_ was confirmed by many experiments, observing the undercooling versus the overheating rates of liquid elements and CoB alloys [28,29]. This nucleation temperature could have, for consequence, the possible existence of a second melting temperature of growth nuclei above T_m_ and of their homogeneous nucleation at temperatures weaker than θ_n+_.

The coefficient ε_ls_ of the initial liquid called liquid 1 is a quadratic function of θ in Equation (8) [6]:

(8) $$\varepsilon_{\mathrm{ls}}=\varepsilon_{\mathrm{ls}0}(1-\theta^{2}/\theta_{0m}^{2})$$

where θ_0m_ is the Vogel–Fulcher–Tammann-reduced temperature leading to ε_ls_ = 0 for θ = θ_0m_, the VFT temperature T_0m_ of many fragile liquids being equal to ≌0.77 T_g_. This quasi-universal value is known for numerous liquids including atactic polymers [30,31].

New liquid states are obtained for θ = θ_n+_ = ε_ls_ and θ = θ_n−_ = (ε_ls_ − 2)/3 with Equation (7). The reduced nucleation temperatures θ_n−_ are solutions of the quadratic Equation (9):

(9) $$\varepsilon_{\mathrm{ls}0}\theta_{n-}^{2}/\theta_{0m}^{2}+3\theta_{n-}+2-\varepsilon_{\mathrm{ls}0}=0$$

There is a minimum value of ε_ls0_ plotted as function of θ_0m_ using (8) and θ_n−_ = (ε_ls_ − 2)/3, determining the relation (10) between θ^2^_0m_ and ε_ls0_ for which the two solutions of (9) are equal in the two fragile liquids [8,32]. These values defined the temperature where the surface energy was minimum and θ^2^_0m_ and ε_ls0_ obeyed Equations (10) and (11):

(10) $$\theta_{0m}^{2}=\frac{8}{9}\varepsilon_{\mathrm{ls}0}-\frac{4}{9}\varepsilon_{\mathrm{ls}0}^{2},$$

(11) $$\varepsilon_{\mathrm{ls}}\left(\theta =0\right)=\varepsilon_{\mathrm{ls}0}=1.5\theta_{n-}+2={a\theta }_{g}+2$$

The value a = 1 in the Equation (10) leads to T_0m_ = 0.769 × T_g_ in agreement with many experimental values [9].

All melts and even liquid elements underwent, in addition, a glass transition because another liquid 2 existed characterized by an enthalpy coefficient ε_gs_ given by Equation (12), inducing an enthalpy change from that of liquid 1 at the thermodynamic transition at T_g_ [7,9,32]:

(12) $$\varepsilon_{\mathrm{gs}}=\varepsilon_{\mathrm{gs}0}(1-\theta^{2}/\theta_{0g}^{2})$$

(13) $$\theta_{0g}^{2}=\frac{8}{9}\varepsilon_{\mathrm{gs}0}-\frac{4}{9}\varepsilon_{\mathrm{gs}0}^{2}$$

(14) $$\varepsilon_{\mathrm{gs}}\left(\theta =0\right)=\varepsilon_{\mathrm{gs}0}=1.5\theta_{n-}+2=1.5\theta_{g}+2$$

The difference Δε_lg_ in the Equation (15) between the coefficients ε_ls_ and ε_gs_ determines the phase 3 enthalpy when the quenched liquid escapes crystallization:

(15) Δεlg(θ)=εls−εgs=εls0−εgs0+Δε−θ2(εls0θ0m2−εgs0θ0g2)

The coefficient $\Delta \varepsilon_{\mathrm{lg}}\left(\theta \right)\;$ defined a new liquid phase called phase 3 undergoing a hidden phase transition below T_g_ and a visible one at θ_n+_, occurring for $\Delta \varepsilon_{\mathrm{lg}}\left(\theta \right)$ = θ_n+_, as shown by Equation (7). This transition was accompanied by an exothermic latent heat equal to $\Delta \varepsilon_{\mathrm{lg}}\left(\theta \right)\times \Delta H_{m}$ corresponding to about 15% of the melting heat [18]. Phase 3 was detected for the first time in supercooled water and associated with glacial phase formation [33,34,35] and recently appears as being associated with configuron formation [13,14,15,16,17,18]. The concept of configurons was initially proposed for materials with covalent bonds which can be either intact or broken [36]; then, it was extended to other systems including metallic systems based on ideas of Egami on bonds between nearest atoms in metals [16,37]. Thus, it is generically assumed that the set of bonds in condensed matter has two states; namely, the ground state corresponding to unbroken bonds and the excited state corresponding to broken bonds. The set of bonds in condensed matter is described in such a way by the statistics of a two-level system [38,39] which are separated by the energy interval G_d_. The two approaches converge because the Gibbs free energy of phase 3 is equal to G_d_. Phase 3 is assumed to be the configuron phase which is preserved above T_m_ in a liquid with medium-range order up to a temperature T_n+_. Both transition temperatures T_g_ and T_n+_ are accompanied by enthalpy or entropy changes of phase 3 and are predicted in many cases: Annealing above and below T_g_, vapor deposition, formation of glacial and quasi-crystalline phases in perfect agreement with experiments. Any transformation of phase 3 changes the initial liquid enthalpy and rejuvenation at T_g_ < T < T_n+_ does not lead to the enthalpy of the initial liquid [18].

Our new publication here was devoted to the simplest case where ultrastable glass and glacial phase are not formed. The value of θ_n+_ was maximum in this case because all transformations below T_g_ and T_m_ modified the liquid state and decrease θ_n+_ [19].

The heat capacity jump at T_g_ was equal to 1.5 × ΔH_m_/T_m_ in polymers as shown in 1960 by Wunderlich [40] and confirmed for many molecular glasses [9] (where ΔH_m_/T_m_ = ΔS_m_ is the crystal melting entropy). The contribution of the undercooled liquid to the total heat capacity per mole is given by (16) using d$\Delta \varepsilon_{\mathrm{lg}}\left(\theta \right)/\mathrm{dT}$:

(16) ΔCp(T)=Cp(liq)−Cp(cryst)=2(T−Tm)Tm2(ΔHm)(εls0θ0m2−εgs0θ0g2)

## 3. Exothermic or Endothermic Heats Observed above the Melting Temperature T_m_

### 3.1. Exothermic Enthalpy Delivered at 688 K in Al_88_Ni_10_Y_2_ for T_m_ = 602 K

**Figure 1:**
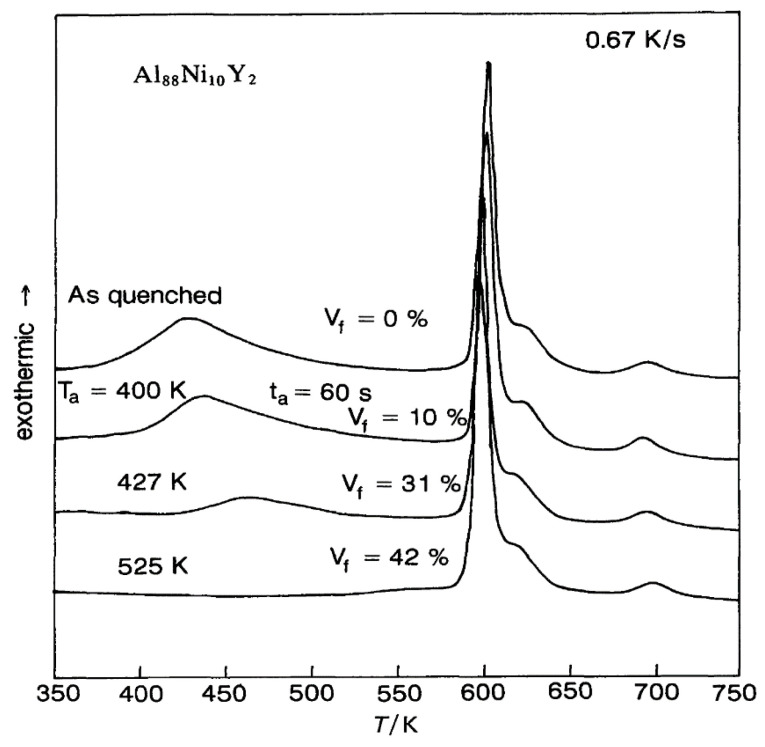
DSC curves measured at 0.67 K/s of an Al_88_Ni_10_Y_2_ amorphous alloy aged for 60 s at different T_a_. Reprinted from ref. [42], Figure 4.

We follow data of ref. [41]. The glass transition occurs at T_g_ = 380 K, and the melting temperature at T_m_ = 602 K. In Figure 1, an annealing of 60 s at T_a_ = 401, 427, and 525 K increases the fraction V_f_ of Al-fcc precipitates up to 0.42 and decreases the volume of the amorphous phase without changing the enthalpy recovery at 688 K measured at 0.67 K/s.

### 3.2. Exothermic Enthalpy Delivered at T_n+_ = 1622 K in (Fe_71.2_B_24_Y_4.8_)_96_Nb_4_

**Figure 2:**
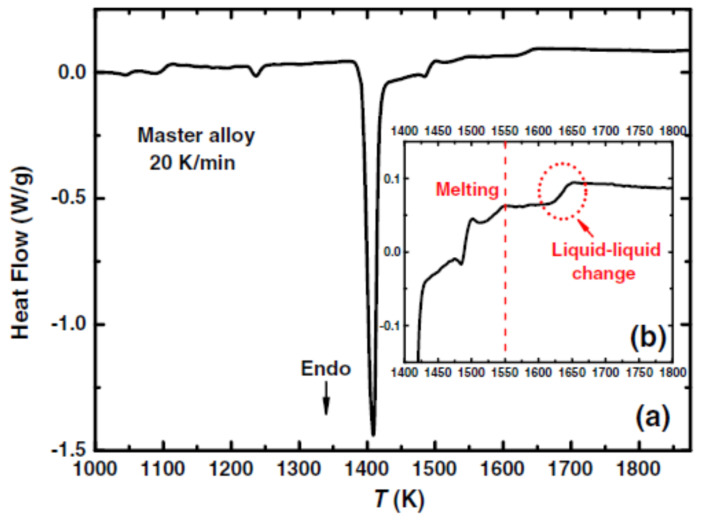
(**a**) High-temperature DSC trace at 0.33 K/s of the master alloy and (**b**) the enlarged version after melting. Reprinted with permission from ref. [42], Figure 7. Copyright 2014 Springer.

We follow data of ref. [42]. The glass transition occurs at T_g_ = 963 K and the melting temperature at T_m_ =1410 K. An enthalpy recovery occurs at 1622 K (Figure 2).

### 3.3. Exothermic Enthalpy Delivered at 1835 K in Ni_77.5_B_22.5_.

We follow data of ref. [24]. The glass transition occurs at T_g_ = 690 K and the melting temperature at T_m_ = 1361 K. The enthalpy recovery temperature is equal to 1835 K.

**Figure 3:**
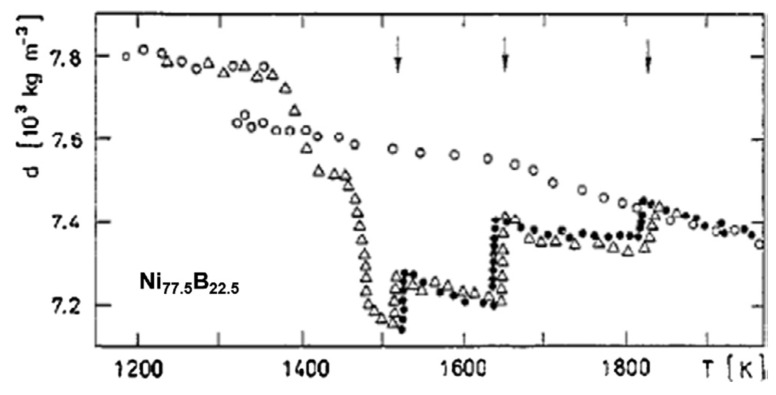
Temperature dependence of the density d of Ni-22.5%B melt at slow heating after melting and time exposition for 5–20 h (•), subsequent cooling (ο), and the second heating after crystallization of the sample and repeated melting (Δ). The arrows show the “critical” temperatures at which the density instability is observed. Reprinted with permission from ref. [24], Copyright 1997 Elsevier.

Deep transformations of eutectic liquid state are observed in Figure 3 by slow heating and aging above the melting temperature which are attributed to the formation of microdomains of 10–100 nm enriched with one of the components with prolonged relaxation time. These microdomains have an influence on the structure and properties of rapidly quenched liquid alloys [24,25]. The enthalpy recovery temperature is here the highest temperature of liquid transformation leading to its homogeneous state. A cooling from 1950 K gives rise to a homogeneous liquid leading to supercooling below T_m_ = 1361 K.

### 3.4. Exothermic Enthalpy Delivered at 1356 K in Cu_47.5_Zr_45.1_Al_7.4_

**Figure 4:**
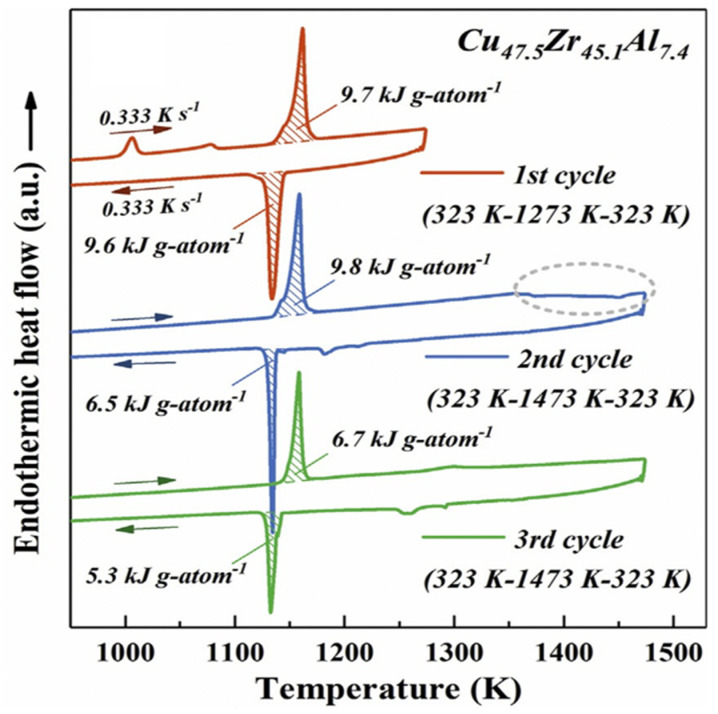
Multiple DTA measurements (0.333 K/s) of Cu47.5 alloy. The first up- and down-scan cycle is well below 1350 K and the last two cycles reach 1473 K. A remarkable exothermic reaction observed at the temperature above 1350 K in the second up-scan curve is marked by a gray dashed circle. Reprinted with permission from ref. [43], Figure 9b, Copyright 2020 Elsevier.

We follow data of ref. [43]. The glass transition occurs at T_g_ = 690 K and the melting temperature at T_m_ =1170 K. An enthalpy recovery occurs at 1356 K (Figure 4).

### 3.5. Endothermic Enthalpy Recovered at 1453–1475 K in a Silicate Liquid

**Figure 5:**
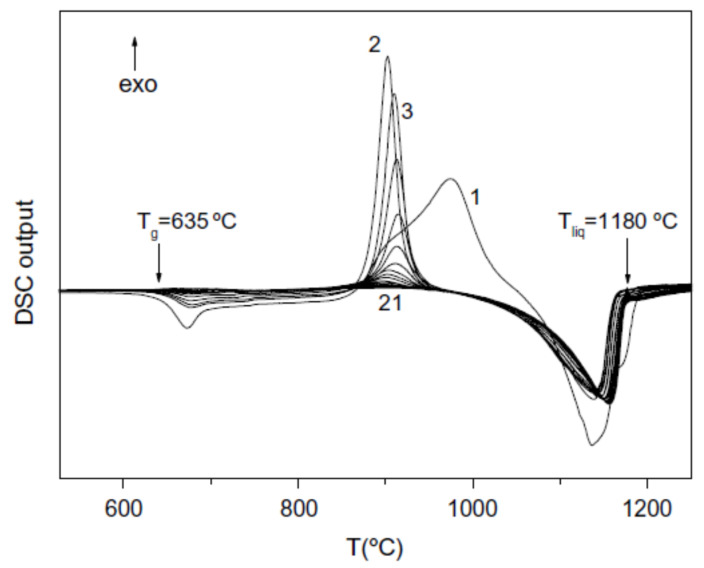
The repeated DSC up-scanning to 1250 °C (T_liq_ + 70 °C) at 0.333 K/s. The numerals next to the graphs represent the order of the DSC up-scans. The measurements are performed in argon at the heating rate 20 °C/min. Reprinted with permission from ref. [44], Figure 2, Copyright 2004 Elsevier.

**Figure 6:**
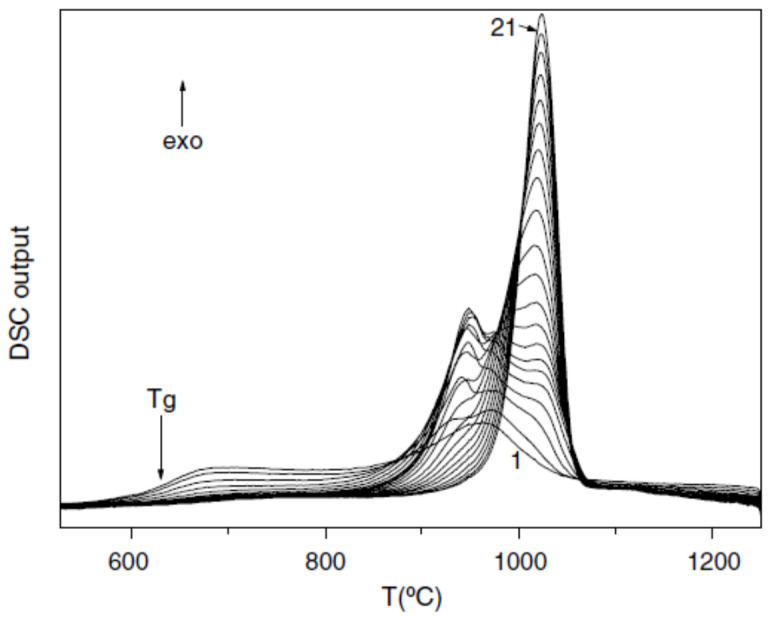
The repeated DSC down-scanning from 1250 °C (T_n+_ + 70°C). The numerals next to the graphs represent the order of the DSC down-scans. The measurements are performed in argon at the cooling rate 20 °C/min. Reprinted with permission from ref. [44], Copyright Elsevier.

We follow data of ref. [44]. The composition was (49.3SiO_2_, 15.6Al_2_O_3_, 1.8TiO_2_, 11.7FeO, 10.4CaO, 6.6MgO, 3.9Na_2_O, and 0.7K_2_O (wt%)). The glass transition occurred at T_g_ = 908 K and the melting temperature at T_m_ = 1313 K. The exothermic latent heat occurred at 1173 K and the amorphous fraction decline with the cycle number from 573 to 1523 K. The melting ex-tended up to 1475 K in Figure 5, and the crystallization temperature Tm occurred at 1313 K in Figure 6. The melting enthalpy recovered between T_m_ and T_n+_ was the same all along the cycles from 2 to 21.

Figure 6 shows that T_m_ = 1313 K. The transition at T_n+_ during continuous cooling at 20 K/mn was no longer sharp and did not have a first-order character. Crystallization occurred at the melting temperature without undercooling, showing that the nuclei were growing between T_n+_ and T_m_ because they were formed above T_m_ by homogenous nucleation accompanied by an enthalpy increase. Phase 3 disappeared above T_n+_ and Δε_lg_ = 0. Crystallization was sharper and sharper during cycling from temperatures higher than T_n+_, showing that the short-range order was enhanced. The enthalpy coefficient Δε_lg_ of phase 3 grew by cooling below T_n+_.

### 3.6. Endothermic Enthalpy Recovered at 1114 K in Zr_41.2_Ti_13.8_Cu_12.5_Ni_10_Be_22.5_ (Vit1)

**Figure 7:**
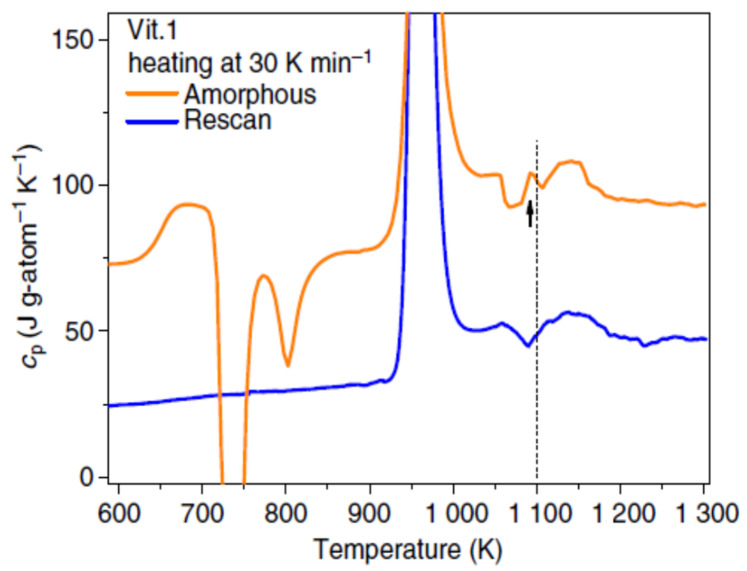
C_p_ measured upon heating at 30 K/min for the amorphous sample (upper) and once-melted crystallized sample (lower) (vertically shifted for clarity). Reprinted from ref. [45], Figure 1b.

We follow data of ref. [45]. The glass transition occurred at T_g_ = 625 K and the melting temperatures at T_sol_ = 965 K and T_liq_ = 1057 K (Figure 7). There was an endothermic enthalpy at T = 1114 K. The heat capacity jump at T_g_ was ΔC_p_ (T_g_) ≌ 21.6 J/K/g-atom. A heat capacity peak of superheated liquid after supercooling was observed during heating around T = 1114 K, accompanied by an endothermic latent heat of about 1100 J/mole. Another transition, observed by viscosity measurements, occurred at 1225 K by heating and subsequent cooling, showing that the liquid became homogeneous above this temperature [46].

Structural changes corresponding to these anomalies were still observed with in-situ synchrotron X-ray-scattering experiments in a contactless environment using an electrostatic levitator (ESL). There was an endothermic liquid–liquid transition at 1114 K during heating reinforced by the symmetrical observation of an exothermic latent heat regarding T_m_ = 965 K and an exothermic structural change around 816 K by supercooling.

### 3.7. Endothermic Enthalpy Recovered at 980–1000 K for T_m_ = 876–881 K in PdNiP Liquid Alloys

**Figure 8:**
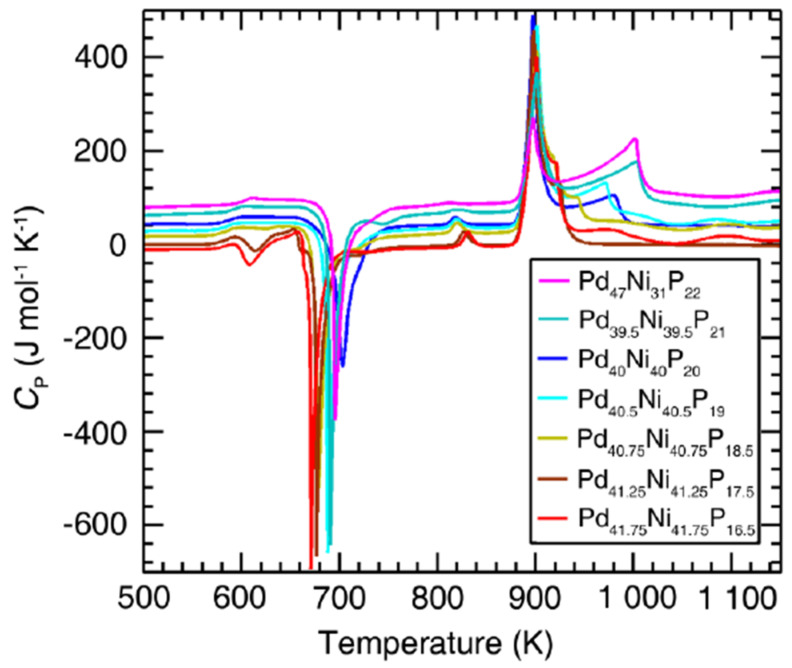
DSC data during heating for Pd-Ni-P metallic glasses. The heating rate is 20 K/min. The curves have been shifted vertically for clarity. Reprinted from ref. [47], Figure S3.

The heat capacities of several PdNiP alloys measured at 20 K/min are represented in Figure 8. The melting temperatures were slowly varying with composition around 880 K and an enthalpy recovery temperature was still observed around 990 K in many liquid alloys. The theoretical predictions for these liquid alloys were limited to the case of Pd_42.5_Ni_42.5_P_15_ [21] presented in Section 6.1 and Section 7.1.

## 4. Predictions of Enthalpy Recovery Temperatures at T_n+_ > T_m_

Equations (10)–(15) were used to calculate the enthalpy coefficients of fragile Liquids 1, 2, and 3 in Section 4.1, Section 4.2, Section 4.4, Section 4.5, and Section 4.6. Liquid Ni_77.5_B_22.5_ in 4.3 being strong, the enthalpy coefficients ε_ls0_ and ε_gs0_ were calculated with (9) for θ_n−_ = θ_g_, θ_0g_^2^ = 1 and θ_0m_^2^ = 4/9.

### 4.1. Exothermic Enthalpy Delivered at T_n+_ = 688 K in Al_88_Ni_10_Y_2_

We follow data of ref. [41]. The enthalpy coefficients of this fragile glass-forming melt were calculated with T_g_ =380 K and T_m_ = 602 K:

(17) $$\mathrm{Liquid}\;1:\;\varepsilon_{\mathrm{ls}}\;\left(\theta \right)=1.63123\left(1-\theta^{2}/0.26736\right)$$

(18) $$\mathrm{Liquid}\;2:\;\epsilon_{\mathrm{gs}}\;\left(\theta \right)=1.44694\left(1-\theta^{2}/0.3557\right)$$

(19) $$\mathrm{Liquid}\;3:\;\Delta \epsilon_{\mathrm{lg}\left(\theta \right)}=0.18439-2.05237\times \theta^{2}$$

The temperature T_n+_ = 688 K was deduced from θ_n+_ = Δε_lg_ (θ_n+_) = 0.14287 [48]. In Figure 1, an exothermic enthalpy peak is observed at 688 K for all samples at 0.67 K/s.

### 4.2. Exothermic Enthalpy Delivered at T_n+_ = 1622 K in (Fe_71.2_B_24_Y_4.8_)_96_Nb_4_

We follow data of ref. [42]. The enthalpy coefficients of this fragile glass-forming melt were calculated with T_g_ = 963 K and T_m_ = 1410 K [48]:

(20) $$\mathrm{Liquid}\;1:\;{\;\varepsilon }_{\mathrm{ls}}\;\left(\theta \right)=1.61206\left(1-\theta^{2}/0.27795\right)$$

(21) $$\mathrm{Liquid}\;2:\;\epsilon_{\mathrm{gs}}\;\left(\theta \right)=1.41609\left(1-\theta^{2}/0.36676\right)$$

(22) $$\mathrm{Liquid}\;3:\;\Delta \epsilon_{\mathrm{lg}\left(\theta \right)}=0.19397-1.93329\times \theta^{2}$$

The temperature T_n+_ = 1622 K was deduced from θ_n+_ = Δε_lg_ (θ_n+_) = 0.1503 [48].

### 4.3. Exothermic Enthalpy Delivered at T_n+_ = 1835 K in Ni_77.5_B_22.5_

We follow data of ref. [24]. The enthalpy coefficients of this strong glass-forming melt were calculated with T_g_ = 690 K and T_m_ = 1410 K:

(23) $$\mathrm{Liquid}\;1:\;\varepsilon_{\mathrm{ls}}\;\left(\theta \right)=1.09891\left(1-\theta^{2}/0.44444\right),$$

(24) $$\mathrm{Liquid}\;2:\;\epsilon_{\mathrm{gs}}\;\left(\theta \right)=0.51347\left(1-\theta^{2}\right)$$

(25) $$\mathrm{Liquid}\;3:\;\;\Delta \epsilon_{\mathrm{lg}\left(\theta \right)}=0.58553-1.958\times \theta^{2}$$

The temperature T_n+_ = 1835 K was deduced from θ_n+_ = Δε_lg_ (θ_n+_) = 0.34808 [48] in agreement with Figure 3.

### 4.4. Exothermic Enthalpy Delivered at T_n+_ = 1356 K in Cu_47.5_Zr_45.1_Al_7.4_

We follow data of ref. [43]. The enthalpy coefficients of this fragile glass-forming melt were calculated from T_g_ = 690 K and T_m_ = 1170 K:

(26) $$\mathrm{Liquid}\;1:\;\varepsilon_{\mathrm{ls}}\;\left(\theta \right)=1.5906\left(1-\theta^{2}/0.28942\right)$$

(27) $$\mathrm{Liquid}\;2:\;\epsilon_{\mathrm{gs}}\;\left(\theta \right)=1.3859\left(1-\theta^{2}/0.37826\right)$$

(28) $$\mathrm{Liquid}\;3:\;\epsilon_{\mathrm{lg}\left(\theta \right)}=0.2047-1.83194\times \theta^{2}$$

The temperature T_n+_ = 1356 K and the recovered enthalpy coefficient Δε_lg_ were deduced from θ_n+_ = Δε_lg_ (θ_n+_) = 0.1586 [48] in agreement with Figure 4. The enthalpy coefficient Δε_lg_ reappeared by homogeneous nucleation below T_n+_ because ε_gs_ (θ_n+_) was weaker than ε_ls_ (θ_n+_) and liquid 1 enthalpy decreased toward that of liquid 2 at slow cooling.

### 4.5. Endothermic Enthalpy Recovered at T_n+_ = 1470 K in a Silicate Liquid

We follow data of ref. [44]. The enthalpy coefficients of this fragile glass-forming melt were calculated with T_g_ = 908 K and T_m_ = 1313 K:

(29) $$\mathrm{Liquid}\;1:\;\varepsilon_{\mathrm{ls}}\left(\theta \right)=1.69155\left(1-\theta^{2}/0.23189\right)$$

(30) $$\mathrm{Liquid}\;2:\;\varepsilon_{\mathrm{gs}}\left(\theta \right)=1.53732\left(1-\theta^{2}/0.31613\right)$$

(31) $$\mathrm{Phase}\;3:\;{\Delta \varepsilon }_{\mathrm{lg}\left(\theta \right)}=0.15473-2.4315\times \theta^{2}$$

The temperature T_n+_ = 1470 K was deduced from θ_n+_ = Δε_lg_ (θ_n+_) = 0.1195 [48] in agreement with Figure 5.

### 4.6. Endothermic Enthalpy Recovered at T_n+_ = 1114 K in Zr_41.2_Ti_13.8_Cu_12.5_Ni_10_Be_22.5_ (Vit1)

We follow data of ref. [45]. The enthalpy coefficients of this fragile glass-forming melt were calculated with T_g_ = 625 K and T_m_ = 965 K [35]:

(32) $$\varepsilon_{\mathrm{ls}}=1.70651\times \left(1-\theta^{2}/0.2226\right)$$

(33) $$\varepsilon_{\mathrm{gs}}=1.4715\times \left(1-\theta^{2}/0.34564\right)$$

(34) $$\;\;\;\;\;\;\;\Delta \varepsilon_{\mathrm{lg}}=0.23501-3.409\times \theta^{2}.$$

The temperature T_n+_ = 1114 K was deduced from θ_n+_ = Δε_lg_ (θ_n+_) = 0.15407 [48] in agreement with Figure 6. The observed double transition was the consequence of the presence in the melt of nuclei, all having the same Gibbs free energy, leading to a homogenous nucleation at 818 and 1114 K as consequence of the quadratic equation of Δε_lg_ (θ_n+_) = θ_n+_. The ordered liquid was rebuilt at T_n+_ = 818 K during cooling from 1350 K with the formation in the no-man’s land of new superclusters, building a vitreous solid phase at T_g_ resulting of the bond number divergence. The hysteresis of viscosity disappeared at about 1225 K when the liquid is homogeneous [46]. A “colloidal” state was melted above the temperature of viscosity or density branching observed during cooling after heating [24,26,27,46]. Equation (35) was used to calculate the reduced temperature θ_n+_ of glass-forming melt with a glass transition at θ_g_ and obeying (11) with a = 1 [48]:

(35) $$\;\theta_{n+}=-0.38742\times \theta_{g}$$

The liquidus melting temperature T_liq_ = 1057.5 K was deduced from Equation (35) with T_g_ = 625 K and T_n+_ = 1225 K in perfect agreement with the experimental observation of liquidus presented in Figure 7. This finding of a second transition above T_n+_ agreed with the first-order liquid–liquid transitions observed above T_n+_ in Pd_42.5_Ni_42.5_P_15_ and La_50_Al_35_Ni_15_.

## 5. Three Liquid States above the Melting Temperature

**Figure 9:**
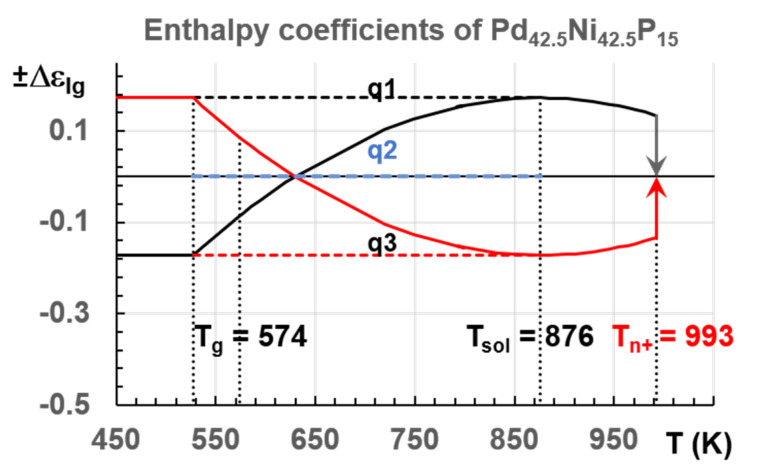
The enthalpy coefficients of Pd_42.5_Ni_42.5_P_15_ versus T (K). T_n+_ = 993 K; Tsolidus = 876 K; T_g_ = 574 K; T_3_ = 527.3 K. The enthalpy coefficients (±Δε_lg_) given by (15) versus T (K). Three quenching lines q1, q2, and q3 along +Δε_lg0_ = 0.17237, 0, and −Δε_lg0_.

The exothermic and endothermic transitions at T_n+_ led to a liquid above T_n+_ with an enthalpy coefficient Δε_lg_ = 0. Two other liquid states existed at T_m_ with enthalpy coefficients equal to ±Δε_lg0_. The melting temperature T_m_ was chosen equal to T_solidus_ in Figure 9. The enthalpy coefficients (±Δε_lg_), defined by (15) and applied to Pd_42.5_Ni_42.5_P_15_ in Figure 9 and in Section 7.1, were related to the enthalpy decrease and increase with temperature of these two quenched liquid states toward that of homogeneous liquid.

The homogenous liquid can be quenched along q2 (Δε_lg0_ = 0) in Figure 9 from above the temperature where the liquid became homogeneous, down to temperatures much weaker than T_g_ [49,50,51]. An enthalpy relaxation at low heating rate, equal to (−Δε_lg0_ × ΔH_m_), built the bonds of phase 3 and led by heating to the temperature where Δε_lg_ = 0 [35]. This slow heating through T_g_ broke the bonds and the liquid enthalpy increases up to (+Δε_lg0_ × ΔH_m_) at T_m_, producing an exothermic enthalpy at T_n+_. These phenomena are observed in Figure 1, Figure 2, Figure 3 and Figure 4.

**Figure 10:**
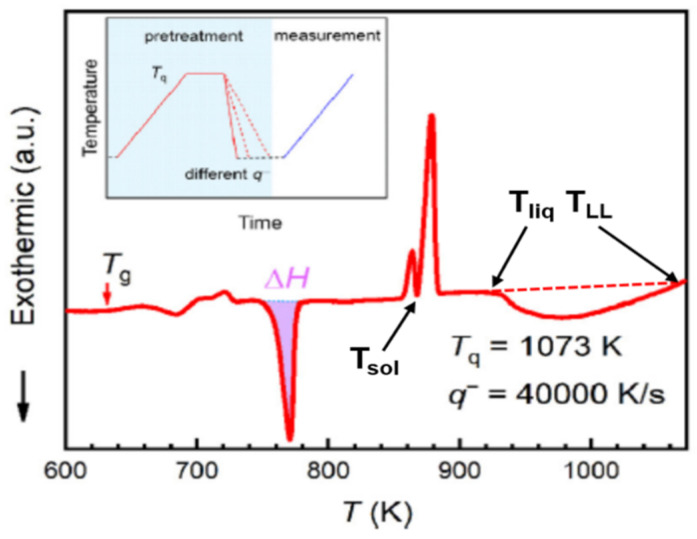
DSC with heating rate of 100 K/s. A typical FDSC heating curve of the sample obtained from T_q_ = 1073 K and q^−^ = 40,000 K/s. The enthalpy of crystallization is denoted as ΔH. The inset shows the temperature protocol of the FDSC experiments. Temperatures T_sol_, T_liq_, and T_LL_ added. Reprinted with permission from ref. [21], Figure 3a, Copyright 2021 Elsevier.

With a much higher heating rate, the enthalpy of bonds, building phase 3, did not have the time to relax below T_g_, and phase 3 was not formed along the thermal path below T_g_ and the latent heat at T_n+_ was not observed for Pd_42.5_Ni_42.5_P_15_ at 100 K/s, as shown in Figure 10 [21]. The liquid being frozen below T_g_ with Δε_lg_ = 0 gave rise to an endothermic enthalpy at T_g_ due to bond breaking and the liquid returned to a homogenous state with Δε_lg0_ = 0 above T_n+_ [10,11,12].

A quench along q1 in Figure 9 from T_m_ < T < T_n+_ with a liquid enthalpy (+Δε_lg0_ × ΔH_m_) at T_m_ led to an amorphous phase with an enthalpy excess (+Δε_lg0_ × ΔH_m_). Phase 3 bonds were built during reheating and they decreased, at a low heating rate, the enthalpy coefficient from (+Δε_lg0_) below T_g_ to (−Δε_lg0_) at T_m_, leading to an endothermic latent heat at T_n+_ corresponding to crystallized nuclei melting at T_n+_.

Starting heating at a very low heating rate from any liquid state led to crystallization and to a liquid enthalpy equal to (−Δε_lg0_ × ΔH_m)_ at T_m_.

A quench from T_m_ < T < T_n+_ along q3 led to the enthalpy of phase 3 with crystallized nuclei being the skeleton of this phase after percolation at T_g_, as shown for plastic crystals. A slow cooling led to crystallization at T_m_ without undercooling [19].

The endothermic and exothermic characters of the transition at T_n+_ were imposed by the initial value of the liquid enthalpy after quenching and by cooling and heating rates.

Homogeneous nucleation in the liquid was expected to depend on the time of aging in the range of temperatures below and close to the homogenization temperature. The first-order liquid–liquid transitions in Pd_42.5_Ni_42.5_P_15_ and La_50_Al_35_Ni_15_ studied by [20,21] combined with our non-classical model of homogeneous nucleation shed light on these new phenomena.

## 6. First-Order Liquid–Liquid Transitions Observed in Pd_42.5_Ni_42.5_P_15_, La_50_Al_35_Ni_15_, and Fe_2_B

We follow data of ref. [21] for Pd_42.5_Ni_42.5_P_15_, of ref. [20] for La_50_Al_35_Ni_15_ and of ref. [24] for Fe_2_B.

### 6.1. Pd_42.5_Ni_42.5_P_15_

#### 6.1.1. Fast Differential Scanning Calorimetry at 100 K/s

The fast differential scanning calorimetry (FDSC) heating curve at 100 K/s represented in Figure 10 and reproduced from [21] was used to determine the solidus and liquidus temperatures T_sol_ = 876 and T_liq_ = 926.5 K. A first-order liquid–liquid transition was observed at T_LL_ = 1063 K. The sample was previously cooled from 1073 K at 40,000 K/s down to room temperature and reheated up to 1073 K, which was a temperature higher than the first-order transition observed at T_LL_.

#### 6.1.2. Melting Transition Observed at 993 K above the Solidus Temperature T_so_l = 876 K of Pd_42.5_Ni_42.5_P_15_

**Figure 11:**
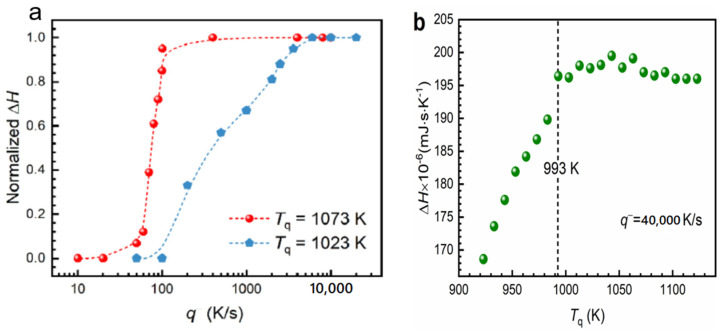
The content of amorphous phase as a function of cooling rate q− of the samples obtained from T_q_ = 1073 and 1023 K, respectively (**a**). The crystallized fraction characterized by ΔH is evaluated by FDSC as shown in Figure 10b. Area of the second exothermic peak ΔH as a function of T_q_ as shown in Figure 10. (**b**) Area of the exothermic peak Δ*H* as a function of *T*_q_ with a cooling rate of 40,000 K/s. Reprinted with permission from ref. [21], Figure 3b and Figure S1, Copyright 2021 Elsevier.

The samples were quenched from T_q_ to room temperature at a cooling rate of q^−^ = 40,000 K/s and reheated at 100 K/s up to T_q_, as shown in Figure 11b [21]. There was no nucleation when cooling started from 1073 K for q > 70 K/s, while crystallization occurred for q < 7000 K/s when cooling started from 1023 K as shown in Figure 11a. The area of the crystallization peak occurring around T = 770 K in Figure 10 was plotted versus T_q_ in Figure 11b. The temperature T = 993 K was viewed by the authors as a liquidus temperature which was, in fact, equal to 926.5 K, as shown in Figure 9.

#### 6.1.3. First-Order Transition Observed by ^31^P Nuclear Magnetic Resonance (NMR)

**Figure 12:**
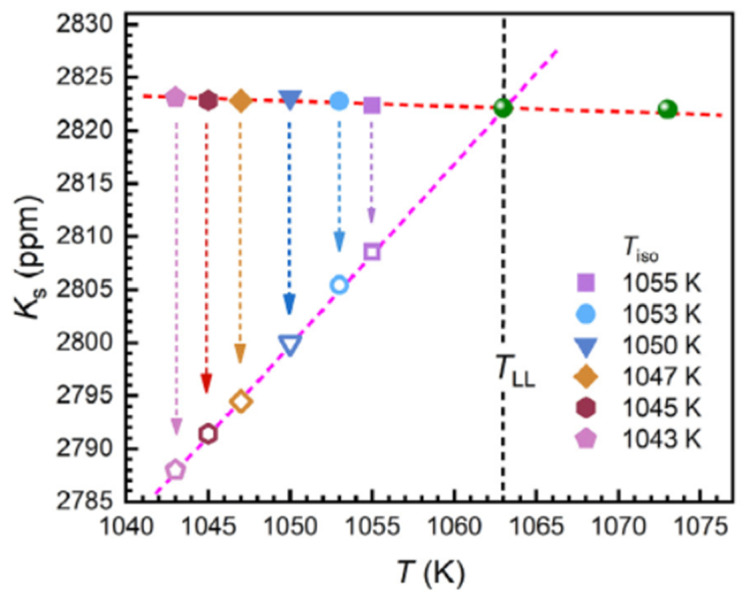
The changes of the Knight shift K_s_ at different undercooled temperatures T_iso_ after quenching the melt from 1173 K. The solid and open symbols represent the initial and equilibrium K_s_, respectively. Reprinted with permission from ref. [21], Figure 1c, Copyright 2021 Elsevier.

^31^P NMR was used to characterize the LLT at T_LL_ = 1063 K above T_n+_ = 993 K. The liquid alloy was first heated to 1293 K for homogenization during 30 min, and then cooled step by step to 1043 K. NMR spectra were taken isothermally after equilibrating the liquid at 1293 K at each step. The Knight shift (K_s_) was determined by the ensemble average of local magnetic field around ^31^P nuclei, sensitive to the changes in structure, plotted in Figure 12 as a function of temperature [21]. (K_s_) varied linearly above 1063 K with a slope increase of 1.76 ppm/K below 1063 K, indicating a change in the P-centered local structures at this temperature. This change was viewed as a first-order liquid–liquid transition (LLT) analogous to that observed in La_50_Al_35_Ni_15_ where a second change of K_s_ in this new liquid state was observed at lower temperatures attributed to the hysteresis of the transition [20]_._

### 6.2. La_50_Al_35_Ni_15_

**Figure 13:**
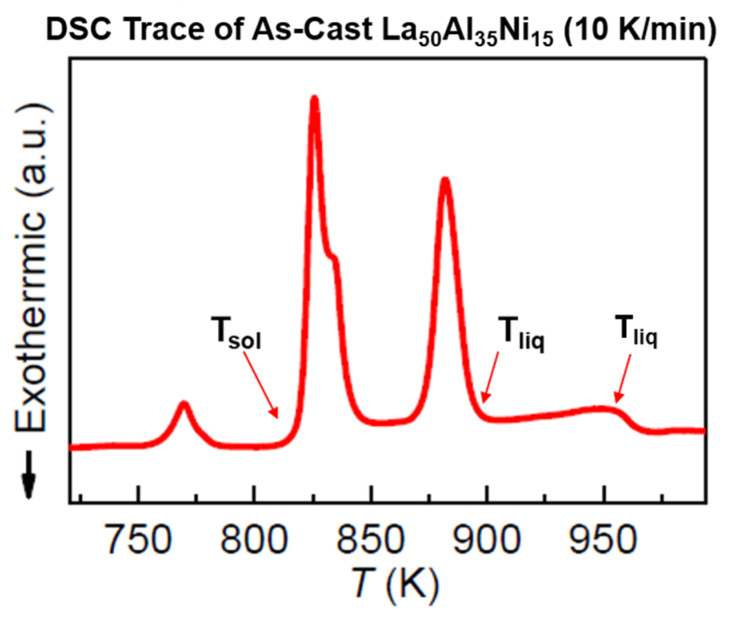
DSC trace of as-cast La_50_Al_35_Ni_15_ BMG. The DSC curve obtained at a heating rate of 10 K/min. Liquidus temperature (T_liq_) indicated by red arrows. The two liquidus temperatures (T_n+_) and solidus temperature (T_sol_) are added. Reprinted from ref. [20], Figure S1.

**Figure 14:**
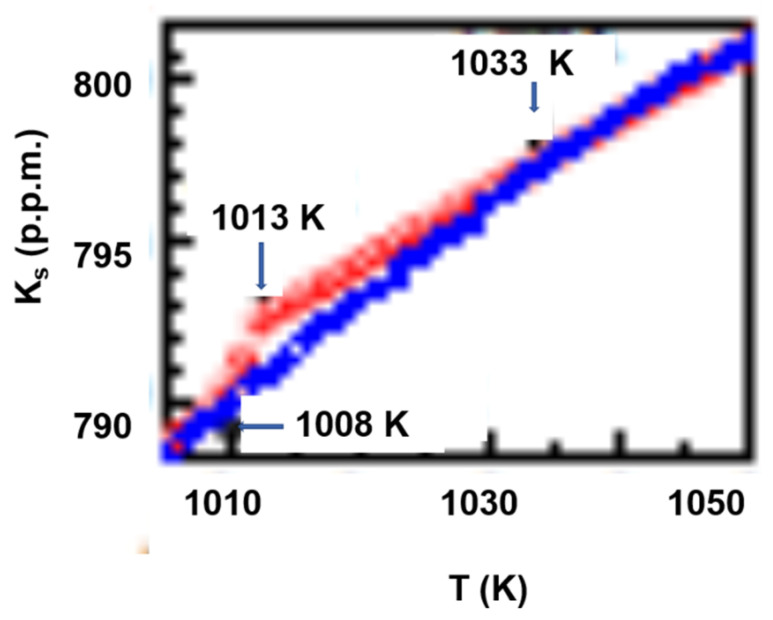
Temperature dependence of ^27^Al knight shift K_s_ during continuous cooling and reheating in the temperature interval of 973–1143 K. The upper red dashed line represents the fitting curves of K_s_ versus T with a slope of 0.22 ppm/K and the lowest blue dashed line represents the fitting curve with a slope of 0.33 p.p.m./K. The two fitting curves intersect at 1033 K. Three characteristic temperatures indicated by black arrows 1033, 1013, and 1008 K. Reprinted from ref. [20], Figure 1b.

This melt was characterized by T_g_ = 528 K, T_sol_ = 877.6 K, and T_liq_ = 892 K, as shown in Figure 13 ([20] Figure S1). A second liquidus temperature was found at 950 K. The temperature T_LL_, observed at 1033 K by measuring the ^27^Al Knight shift by RMN, is viewed as a first-order LLT in Figure 14. A phenomenon analogous to hysteresis led to a second transition at 1013 K.

### 6.3. Fe_2_B

**Figure 15:**
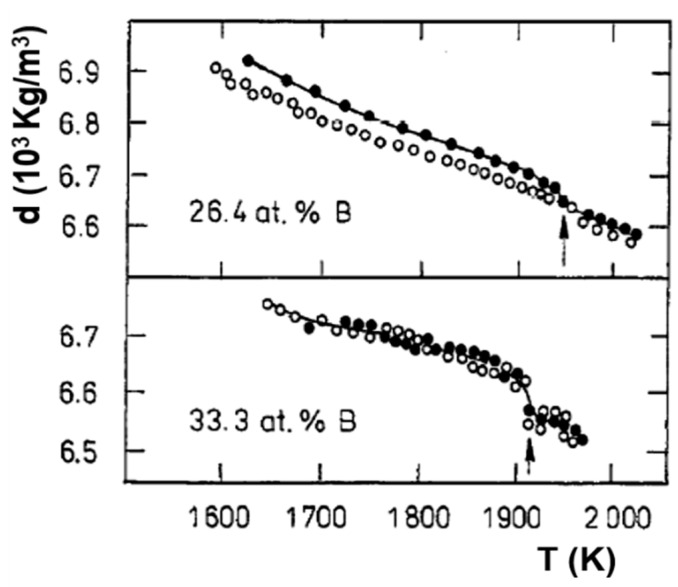
Temperature dependence of the density d of Fe-26.4 at %B and Fe-33.3 at %B melts at heating after melting (•) and subsequent cooling (ο). Arrow shows the anomaly linked with structural transformation in a liquid compound. Reprinted with permission from ref. [24], Figure 8, Copyright 1997 Elsevier.

The vitreous state of this compound was obtained by mechanical alloying [52]. The first-order transition occurs at T_LL_ = 1915 K in Figure 15 with a melting temperature of 1662 K [24].

## 7. Predictions of First-Order Transition Temperatures by Homogenous Nucleation in Pd_42.5_Ni_42.5_P_15_, La_50_Al_35_Ni_15_ and Fe_2_B Melts

These first-order transitions were observed at T_LL_ at very low cooling rates or by isothermal annealing between the melting temperature and T_LL_. The homogeneous liquid state characterized by Δε_lg0_ = 0 was stable during cooling in Figure 3 while the first-order transitions were reversible in Figure 15. The two melting temperatures T_sol_ and T_liq_ of non-congruent materials led to two nucleation temperatures T_n+_.

### 7.1. Predictions of Transitions in Pd_42.5_Ni_42.5_P_15_ Melt

The temperature 993 K in Figure 11b was viewed by [21] as a liquidus temperature which was, in fact, equal to 926.5 K, as shown in Figure 10. The reduction of the enthalpy recovered by crystallization at 770 K occurred for T_q_ < 993 K, as shown in Figure 11b. The crystallization enthalpy at 770 K was continuously reduced without exothermic enthalpy jump equal 0.13357 × 197 = 26 in Figure 11b at 993 K. The mean-range order accompanied by exothermic enthalpy progressively reappeared by homogeneous nucleation in the liquid heated during 30 s at each temperature T_q_ and was completely formed at T_liq_ = 926.5 K because the enthalpy decrease was equal to −13.4 % at this temperature. The residual configurons melted at T_n+_ = 993 K using (35) (θ_n+_ = Δε_lg_ (θ_n+_) = 0.13357), T_m_ = 876 K, and T_g_ = 574 K. This value of T_g_ agreed with measurements of heat capacity of melts with similar compositions [47]. The enthalpy coefficients of Pd_42.5_Ni_42.5_P_15_ for the liquidus and solidus liquid states were given in Equations (36–38) using Equations (10–16):

For T_sol_ = 876 K and T_g_ = 574 K

(36) $$\mathrm{Liquid}\;1:\;\varepsilon_{\mathrm{ls}}\left(\theta \right)=1.65525\left(1-\theta^{2}/0.25362\right)$$

(37) $$\mathrm{Liquid}\;2:\;\varepsilon_{\mathrm{gs}}\left(\theta \right)=1.48288\left(1-\theta^{2}/0.34081\right)$$

(38) $$\mathrm{Phase}\;3:\;{\Delta \varepsilon }_{\mathrm{lg}\left(\theta \right)}=0.17237-2.1755\times \theta^{2}$$

For T_Liq_ = 926.45 K and T_g_ = 574 K

(39) $$\mathrm{Liquid}\;1:\;\varepsilon_{\mathrm{ls}}\left(\theta \right)=1.61957\left(1-\theta^{2}/0.27384\right)$$

(40) $$\mathrm{Liquid}\;2:\;\varepsilon_{\mathrm{gs}}\left(\theta \right)=1.42935\left(1-\theta^{2}/0.36251\right)$$

(41) $$\mathrm{Phase}\;3:\;{\Delta \varepsilon }_{\mathrm{lg}\left(\theta \right)}=0.19022-1.97146\times \theta^{2}$$

Applying Equation (35) led to T_n+_ = T_LL_ = 1063 K in perfect agreement with Figure 12. From our analysis_,_ a second change of K_s_ occurred by homogeneous nucleation in Pd_42.5_Ni_42.5_P_15_ at T_n+_ = 993 K. This transition was not only due to the hysteresis of a first-order transition because there were two homogeneous nucleation temperatures as shown in Figure 12. This point was still confirmed in 7.2 devoted to La_50_Al_35_Ni_15_, where the changes of K_s_ occurred for two values of T_n+_ because there were, in these non-congruent liquid compounds, two solid–liquid transitions characterized by solidus and liquidus temperatures.

The temperature T_n+_ = T_LL_ = 1063 K corresponded to the temperature of homogenous nucleation of colloids containing critical numbers n_c_ of atoms with n_c_ given by Equation (42) (see [9], Equation (48)):

(42) $$n_{c}=\frac{8N_{A}k_{B}{\left(1+\Delta \varepsilon_{\mathrm{lg}}\right)}^{3}}{27\Delta S_{m}{\left(\Delta \varepsilon_{\mathrm{lg}}\right)}^{3}}\ln(K)$$

where N_A_ is the Avogadro number, k_B_ the Boltzmann constant, ΔS_m_ the melting entropy, and LnK ≌ 90 [5]. With Δε_lg_ = 0.13356 and ΔS_m_ = 8.76 J/g-atom [47], n_c_ = 15522 at the temperature T_n+_ = 993 K. With Δε_lg_ = 0.14739 and ΔS_m_ = 8.76 J/g-atom, n_c_ = 11977 at the temperature T_n+_ = 1063 K. Critical numbers n_c_, still larger, were observed in Pb-Bi liquid alloys below the temperature of liquid homogenization [27]. The number of atoms inside an elementary superatom in the homogenous liquid above 1063 K was equal to 135, with Δε_lg_ (θ_n+_) replaced in (42) by ε_gs_ (θ_n+_) = 1.40526 in (42) using Equations (39) and (40). The homogenous nucleation time τ (s) for temperatures 1043 < T < 1063 K was following Equation (43) (see Figure 1d in ref. [21]):

(43) $$\tau \;\left(s\right)=5.9\times 10^{-3}{\left(\frac{1063}{T}-1\right)}^{2.18}$$

which led by extrapolation to τ ≌ 1.9 s at T_n+_ = 993 K.

There was no growth nucleus inducing crystallization after quenching from the temperature T = 1073 K which was higher than T_LL_ = 1063 K as shown in Figure 11a [21]. New growth nuclei were added when the melt was quenched from 1023 K, a temperature higher than T_n+_ = 993 K and much higher than T_sol_. Consequently, new denser nuclei growing from the colloidal state were added by homogeneous nucleation at 1023 K above 993 K. The transition at 993 K after cooling from 1073 K was due to the internal and external bond formation between colloids below 1063 K [18]. This observation agreed with the growth of n_c_ from 11977 to 15522 between 1063 and 993 K. The transitions observed by NMR below 1063 K involved all ^31^P atoms and corresponded to the colloid formation through the relaxation time decrease [23]. The first-order character of this transition was observed at each step of isothermal annealing below 1063 K. The breaking of bonds inside and outside colloids occurred at the lowest temperature T_n+_ during heating [18], while at the highest T_n+_, a transition from colloidal state to a new homogeneous state made of elementary superatoms only organized by short-range order appeared.

**Figure 16:**
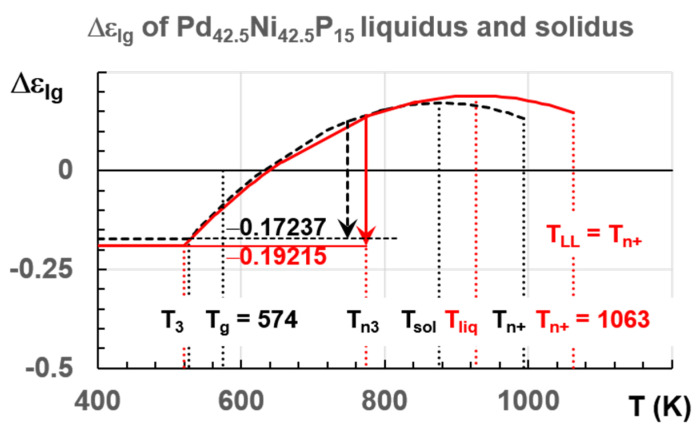
Enthalpy coefficients of liquidus and solidus melts versus T (K). T_liq_ = 926.5 K, T_n+_ = 1063 K, T_sol_ = 876 K, and its T_n+_ = 993 K. Crystallization at the nucleation temperature T_3_ = 748.4 K of phase 3 in solidus melt instead of T_3_ = 774.2 K in liquidus melt. In the liquidus melt, T_LL_ = T_n+_ = 1063 K.

The enthalpy coefficients (−Δε_lg0_) of phase 3 equal to those of configurons are represented in Figure 16 as a function of the temperature T (K) for T_sol_ and T_liq_ using Equations (38) and (41). The crystallization temperature occurred at the reentrant formation temperature of ultrastable glass with its enthalpy equal to −Δε_lg0_. This nucleation temperature opened the door to crystallization [19].

### 7.2. Predictions of First-Order Transitions in La_50_Al_35_Ni_15_ Glass-Forming Melt

**Figure 17:**
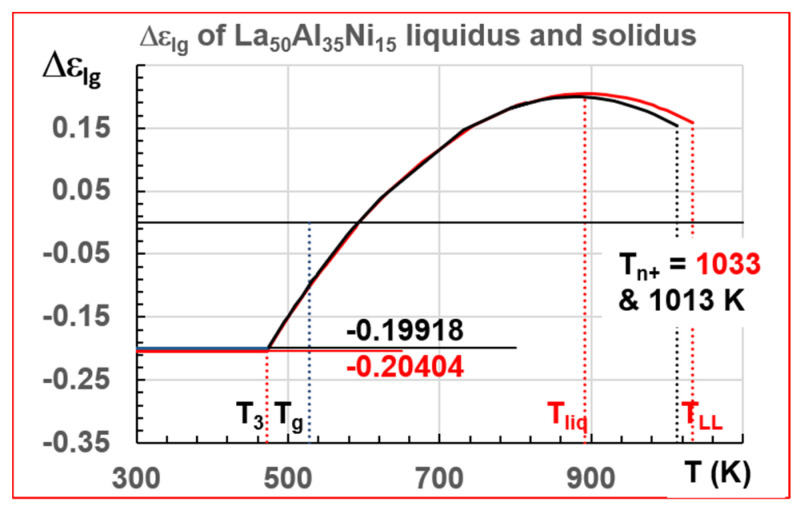
La_50_Al_35_Ni15 enthalpy coefficients of liquidus and solidus melts. T_sol_ = 877.6 K; T_n+_ = 1013 K; T_liq_ = 892 K; T_n+_ = T_LL_ = 1033 K. The enthalpy coefficients of ultrastable phase 3 are (−0.19918) for the solidus and (−0.20404) for the liquidus. The two melts have the same T_g_ = 574 K.

We follow data of ref. [20]. The phase 3 enthalpy coefficients of La_50_Al_35_Ni_15_ for the liquidus and solidus liquids were given in Equations (44)–(49) for T_sol_ = 877.6 K, T_liq_ = 892 K, and T_g_ =528 K, and are represented in Figure 17.

For T_Liq_ = 892 and T_g_ = 528 K:

(44) $$\mathrm{Liquid}\;1:\;{\;\varepsilon }_{\mathrm{ls}}\left(\theta \right)=1.66143\left(1-\theta^{2}/0.25000\right)$$

(45) $$\mathrm{Liquid}\;2:\;\varepsilon_{\mathrm{gs}}\left(\theta \right)=1.42935\left(1-\theta^{2}/0.33679\right)$$

(46) $$\mathrm{Phase}\;3:\;{\Delta \varepsilon }_{\mathrm{lg}\left(\theta \right)}=0.20404-2.2516\times \theta^{2}$$

For T_sol_ = 877.6 and T_g_ = 528 K:

(47) $$\mathrm{Liquid}\;1:\;\varepsilon_{\mathrm{ls}}\left(\theta \right)=1.60164\left(1-\theta^{2}/0.28357\right)$$

(48) $$\mathrm{Liquid}\;2:\;\varepsilon_{\mathrm{gs}}\left(\theta \right)=1.40246\left(1-\theta^{2}/0.37246\right)$$

(49) $$\mathrm{Phase}\;3:\;\Delta \varepsilon_{\mathrm{lg}\left(\theta \right)}=0.19218-1.88273\times \theta^{2}$$

### 7.3. Predictions of Glass Transition Temperature of Fe_2_B Melt

**Figure 18:**
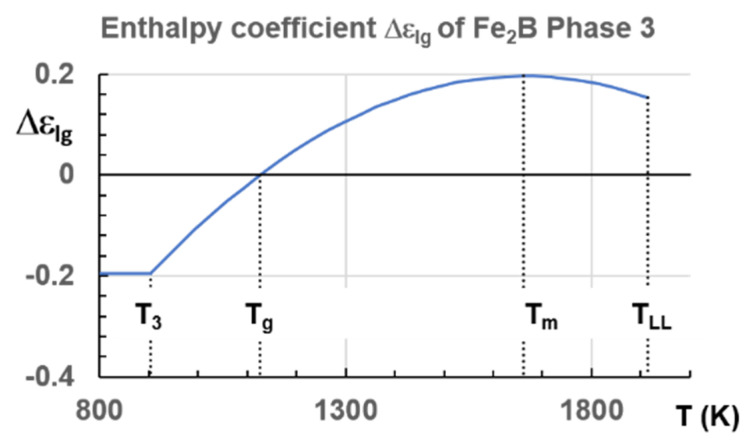
Enthalpy coefficient Δε_lg_ (T) of Fe_2_B Phase 3 and configurons. T_3_ = 903 K, T_g_ = 1125.7 K, T_m_ = 1662 K, T_n+_ = T_LL_ = 1915 K.

The enthalpy coefficients of the strong liquid Fe_2_B were calculated with Equations (9) and (35), T_m_ = 1662 K and T_n+_ = T_LL_ = 1915 K, given in Equations (50)–(52). Phase 3 is represented in Figure 18.

For T_m_ = 1662 K and T_n+_ = T_LL_ = 1915 K, T_g_ = 1125.7 K:

(50) $$\mathrm{Liquid}\;1:\;\;\varepsilon_{\mathrm{ls}}\left(\theta \right)=1.60164\left(1-\theta^{2}\times 2.25\right)$$

(51) $$\mathrm{Liquid}\;2:\;\varepsilon_{\mathrm{gs}}\left(\theta \right)=1.1519\left(1-\theta^{2}\right)$$

(52) $$\mathrm{Phase}\;3:\;{\Delta \varepsilon }_{\mathrm{lg}\left(\theta \right)}=0.19579-1.8804\times \theta^{2}$$

### 7.4. One Liquid–Liquid Transition at T_n+_ = T_LL_ in Congruent Materials and Two in the Others

A first-order transition occurred at T_LL_ due to the formation by cooling of colloidal state assembling elementary superatoms composed of tenths atoms bounded by short-range interactions, leading to colloids containing thousands of atoms. In the case of congruent materials, only one liquid–liquid transition was expected. The lowest and the highest temperatures T_n+_ were equal and T_n+_ is a first-order transition temperature equal to T_LL_. This is the case for Fe_2_B.

These colloids were similar atom clouds containing a magic atom number of atoms because they were melted by homogeneous nucleation instead of surface melting. They had a maximum radius for which their Gibbs free energy was smaller or equal to that of the melt [53].

There were two liquid–liquid transitions above the solidus and liquidus temperatures T_sol_ and T_liq_ in non-congruent materials, leading to two temperatures T_n+_. The highest one was equal to T_LL_ and related to T_liq_. Above T_LL_, the liquid was homogeneous and atoms were only submitted to short-range order in tiny superatoms. The lowest (T_n+_) was related to T_sol_ and was the temperature where coupling between elementary superatoms started during cooling and led to bond percolation at T_g_. The lowest one was a second-order phase transition where the residual configurons were melted during heating, involving 15% of the sample volume.

A melt was only rejuvenated above T_n+_ because all colloids and superatoms were disconnected.

## 8. Perspectives: Mpemba Effect and Bonding-Antibonding of Superatoms

### 8.1. Mpemba Effect and Its Inverse Relation to the Existence of Three Liquid States above the Melting Temperature

The Mpemba effect is described by a shorter time needed to crystallize a hot water system than to crystallize the same colder water system cooled down from initial lower temperatures [54]. This phenomenon was documented by Aristotle 2300 years ago [55]. The melting enthalpy of ice was ΔH_m_ = 334 J/g with a specific heat of 4.18 J/g. Starting from a hot homogenous water, the exothermic enthalpy of formation of mean-range order below T_n+_ = 295.3 K (22.1 °C) [34] was progressively equal to −0.0818 ×334 = −27.3 J/g by homogenous nucleation during slow cooling through T_n+_. The value of T_n+_ in water was confirmed by numerical simulations of the melting temperature of an ultrathin layer of hexagonal ice [19,56,57,58]. The water enthalpy variation being equal to 92 J/g from 22.1 to 0 °C, the temperature of 0 °C was quickly attained by the hot system because of the recovery of exothermic enthalpy. The cold water had no more available exothermic enthalpy because the formation of mean range order was much older in this water. Cooling this liquid took much more time.

The latent heat, expected at T_n+_ = 22.1 °C, was not observed up to now, while Mpemba and Osborne observed this effect with a slow cooling rate of 0.01 K/s. The window of nucleation was very narrow in congruent materials because the temperature T_n+_ was unique instead of extending between the two T_n+_ temperatures of non-congruent substances as shown in Figure 13. At a too-high cooling rate, the liquid state, with Δε_lg_ = 0, free of any growth nucleus, remained stable and showed undercooling. The homogeneous liquid state was stable when it escaped the formation of colloidal state at T_n+_. Figure 3 showed this phenomenon in Fe_77.5_B_22.5_. On the contrary, the transition of Fe_2_B at T_n+_ = 1915 K had a first-order character (see Figure 15) Nucleus formation started from the colloidal state and was expected to be formed at a low cooling rate.

Using the theory of nonequilibrium thermodynamics, Lu and Raz predicted a similar anomalous behavior with heating using a three-state model that we had here for all melts [59]. A cold liquid, with an enthalpy equal to (+Δε_lg0_ × ΔH_m_), obtained after building bonds below T_g_, would develop an exothermic latent heat at T_n+_ during heating, while a warmer liquid with an enthalpy equal to (−Δε_lg0_ × ΔH_m_) would need an endothermic enthalpy to melt its mean-range order.

The Mpemba effect and its inverse effect can be extended to many systems [59,60] and we showed that these phenomena could exist in all melts. Moreover, we assumed that analogues of Mpemba effects should occur on vitrification of liquids so that glasses would be formed quicker out of hot melts compared with melts cooled down from lower temperatures. All these new events were observable because the transition at T_n+_ was a first-order transition in congruent materials [24].

### 8.2. Three Liquid States Associated with Bonding–Antibonding of Superatoms

In Figure 9, the enthalpy coefficient of phase 3 at T_n+_ was equal to three values +Δε_lg_, 0, and −Δε_lg_ depending on thermal history leading to three liquid states. The glass transitions occurred at the percolation threshold of superclusters, built by homogeneous nucleation, during the first cooling of liquids initially homogeneous. These superclusters survive in overheated colloids after their formation during the first cooling because they were melted at T_n+_ by homogeneous nucleation instead of surface melting at T_m_. These entities were contained in colloids with a magic atom number [61]. Thus, they were melted at T_n+_ when their Gibbs free energy became equal to that of homogeneous liquid [53]. The endothermic and exothermic latent heats revealed the existence of two families of bound molecules which could be attributed to bonding and antibonding of colloids through elementary superatoms. This concept of bonding and antibonding is highly developed in bond chemistry to create new chemical structures. Bonding and antibonding of colloids could lead to higher and lower enthalpies. Two recent examples of research in this field were given [62,63]. The nature built these new superstructures in all overheated melts by homogeneous nucleation. The percolation of these superatoms led T_g_ to a superstructure involving 3D space in 15% of atoms [18].

## 9. Conclusions

Our models of homogeneous nucleation and configurons explained the formation of liquid phases above T_g_ with mean-range order disappearing at a temperature T_n+_ much higher than the melting temperature. This transformation at T_n+_ was a first-order transition in congruent materials such as Fe_2_B and was expected to be observable at a very low cooling rate or by homogeneous nucleation during isotherm annealing below T_n+_. There were two melting temperatures in non-congruent materials called solidus and liquidus temperatures, leading to two temperatures: T_n+_ starting with a unique glass transition temperature T_g_. The first-order liquid–liquid transitions in Pd_42.5_Ni_42.5_P_15_ and La_50_Al_35_Ni_15_ observed with NMR at T_LL_ = 1063 and 1033 K, respectively, occurred at the temperature T_n+_ corresponding to the liquidus temperature of these two alloys. The two other second-order phase transitions, occurring at T_n+_ = 993 and 1013 K respectively, were induced by the solidus temperatures.

The latent heats produced at T_n+_ were exothermic or endothermic. We attributed this phenomenon to the presence of three liquid states at T_n+_, with three enthalpy coefficients depending on the cooling rate and on the starting temperature of quenching. These enthalpies were equal to 0, +ΔH_m_ × Δε_lg_ (T_n+_), and −ΔH_m_ × Δε_lg_ in comparison with that of a homogeneous liquid equal to zero at high temperatures. These liquids, when quenched to temperatures much weaker than T_g_, were characterized by their initial enthalpy at the solidus temperature. The liquid state enthalpy after quenching was (−ΔH_m_ × Δε_lg0_), or (+ΔH_m_ × Δε_lg0_), or 0 depending on its initial value before quenching and on the cooling and heating rates. The enthalpy increased from −ΔH_m_ × Δε_lg0_ and 0 up to +ΔH_m_ × Δε_lg0_ at T_m_ with configuron melting. The enthalpy decreased from +ΔH_m_ × Δε_lg0_ and 0 to −ΔH_m_ × Δε_lg0_ at T_m_, rebuilding the missing bonds. These phenomena were well described by the positive or negative variation ±ΔH_m_ × Δε_lg_ (T_n+_), of enthalpies of bonds and configurons.

Our homogeneous nucleation model above T_m_ still confirmed the formation of colloids between T_n+_ and T_m_ and at slow cooling rate, the growth of cluster-bound colloids inducing crystallization. The temperature T_n+_, congruent materials being unique, was the temperature of homogenization of these melts. The highest temperature T_n+_ = T_LL_ observed in Pd_42.5_Ni_42.5_P_15_ and La_50_Al_35_Ni_15_ was a homogenization temperature of these non-congruent materials. All melts, containing atoms of different nature, were submitted to short-range order inside superatoms, being the elementary bricks building the ordered liquids and glasses.

These colloids and elementary superatoms could not be more precisely described because their magic atom number n_c_, and the associated enthalpy depending on n_c_, were unknown.

Colloids formed by homogeneous nucleation were superatoms containing magic atom numbers which were not totally melted above T_m_ and were fully melted by homogenous nucleation instead of surface melting at the highest temperature T_n+_. They contained a critical atom number n_c_ defined by their Gibbs free energy equal or smaller than that of the homogeneous melt. They gave rise to new molecular entities by bonding and antibonding, as shown by the opposite values of their contribution to the enthalpy at T_n+_. Superstructures of elementary superatoms grew during cooling down to their percolation temperature.

The Mpemba effect and its inverse were easily predicted from this description of materials melting, leading to three stable liquid states above the melting temperature and transitions between them. The transition at T_n+_ may have been not only the temperature where the mean-range order disappeared, but also a first-order transition temperature between two liquid states.

## Data Availability

The data underlying this article will be shared on reasonable request from the corresponding author.
